# Domestication history and genetic changes for the newly evolved flower color in the ornamental plant *Lobularia maritima* (Brassicaceae)

**DOI:** 10.1093/hr/uhae355

**Published:** 2024-12-19

**Authors:** Wenjie Yang, Meng Liu, Landi Feng, Pengfei Jiao, Jiebei Jiang, Li Huang, Jianquan Liu, Jordi López-Pujol, Quanjun Hu

**Affiliations:** Key Laboratory of Bio-Resource and Eco-Environment of Ministry of Education, College of Life Sciences, Sichuan University, Chengdu 610065, China; Key Laboratory of Bio-Resource and Eco-Environment of Ministry of Education, College of Life Sciences, Sichuan University, Chengdu 610065, China; Key Laboratory of Bio-Resource and Eco-Environment of Ministry of Education, College of Life Sciences, Sichuan University, Chengdu 610065, China; Key Laboratory of Bio-Resource and Eco-Environment of Ministry of Education, College of Life Sciences, Sichuan University, Chengdu 610065, China; Key Laboratory of Bio-Resource and Eco-Environment of Ministry of Education, College of Life Sciences, Sichuan University, Chengdu 610065, China; Key Laboratory of Bio-Resource and Eco-Environment of Ministry of Education, College of Life Sciences, Sichuan University, Chengdu 610065, China; Key Laboratory of Bio-Resource and Eco-Environment of Ministry of Education, College of Life Sciences, Sichuan University, Chengdu 610065, China; State Key Laboratory of Grassland AgroEcosystem, College of Ecology, Lanzhou University, Lanzhou 730000, China; Botanical Institute of Barcelona (IBB), CSIC-CMCNB, Barcelona 08038, Spain; Escuela de Ciencias Ambientales, Universidad Espíritu Santo (UEES), Samborondón 091650, Ecuador; Key Laboratory of Bio-Resource and Eco-Environment of Ministry of Education, College of Life Sciences, Sichuan University, Chengdu 610065, China

## Abstract

*Lobularia maritima* (sweet alyssum) is a popular ornamental plant that displays a range of flower colors, particularly white and purple. However, the genetic underpinning and evolutionary history of flower colors have remained unknown. To address this, we performed a *de novo* assembly of a chromosome-level genome for this species and conducted comparative population genomic analyses of both domestic and wild representatives. These analyses revealed distinct genetic clusters corresponding to wild and domestic groups, with further subdivisions based on geographic and phenotypic differences. Importantly, all cultivars originated from a single domestication event within the Tunisia group. One wild group did not contribute genetically to the current cultivars. The new mutations in key gene of the anthocyanin biosynthetic pathway, *PAP1*, that arose following domestication led to the origin of purple flower coloration in the cultivars. Moreover, the contrasting *PAP1* haplotypes in white and purple varieties lead to differential expression of *CHS* and *DFR*, which in turn contributes to the observed flower color differences. These findings provide key insights into the domestication history and genetic regulation of flower color in *L. maritima*, laying the groundwork for future genetic breeding efforts focused on this plant, especially introducing genetic sources from other wild groups.

## Introduction

Flower color is one of the most critical traits in ornamental plants, significantly influencing their aesthetic value and commercial appeal as well as their defense against various biotic and abiotic stresses [1–3]. The underlying genetic variations that contribute to color diversity have been extensively studied across many ornamental species [1, 4–6]. This color diversity is found to be primarily determined by the types and accumulations of anthocyanin and their derivatives [7, 8]. Anthocyanins, a group of flavonoid compounds, are the pigments that produce red, purple, and blue hues in ornamental plants [9, 10]. The anthocyanin biosynthesis starts from phenylalanine and progresses through three phases: phenylpropanoid, flavonoid, and anthocyanin-specific synthesis [1, 8]. Regulation of the structural genes in this process is primarily governed by the MYB-bHLH-WD40 (MBW) transcription complex, comprising MYB and bHLH transcription factors alongside WD40 proteins [11, 12]. The R2R3 MYB protein, production of anthocyanin pigments1 (PAP1), serves as the primary driver of anthocyanin biosynthesis by activating the expression of the corresponding biosynthetic genes [13–16]. Understanding the regulatory mechanisms involving these transcription factors is crucial for comprehending the expression patterns and their roles in determining final flower colors.

*Lobularia maritima* (L.) Desv. (syn.: *Alyssum maritimum* (L.) Lam.), commonly referred to as sweet alyssum or sweet alison, is classified into the Brassicaceae family. The flowers are sweet-smelling, with an aroma reminiscent of honey, and have four rounded petals and four sepals. Native to the Mediterranean Basin [17], wild *L. maritima* typically exhibits white flowers. However, cultivated varieties are now extensively grown worldwide for their ornamental value, displaying a range of colors such as white, pink, lavender, and purple [18–21]. According to reports from the Germplasm Resources Information Network (https://npgsweb.ars-grin.gov/gringlobal/search) and the RHS Plant Finder (https://www.rhs.org.uk/plants), there are over 15 different cultivars of *L. maritima* officially recognized, with more than 30 additional varieties potentially available for purchase from various sources [21]. This color diversity in cultivated varieties highlights the impact of domestication and selective breeding on floral traits [22, 23]. Understanding the relationships among wild populations and how geographic differentiation contributes to their diversity, as well as exploring the genetic causes of different flower colors in cultivated populations, are both crucial for comprehending domestication and evolution of *L. maritima* underlying strong artificial selection.

**Figure 1:**
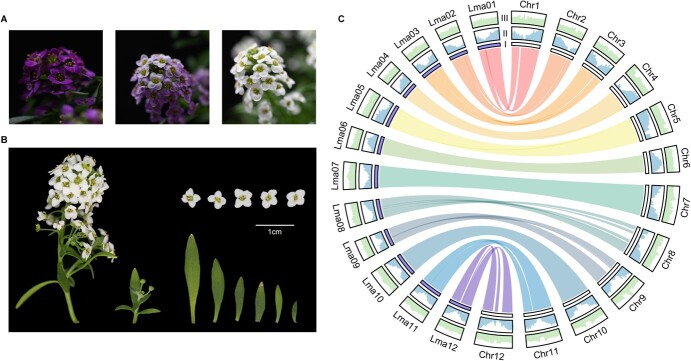
Genomic features and floral traits of *L. maritima*. (A) Photo of different cultivars of *L. maritima*. (B) Photo of the sample used in genome sequencing. (C) Circos plot illustrating the genomic characteristics across various versions of the *L. maritima* genome. (I) The innermost circle depicts the individual chromosomes of each genome. The bars labeled Lma01–12 represent the chromosomes of LmG v1.0, while the bars labeled Chr1–12 represent the chromosomes of LmG v2.0. (II) Gene density. (III) GC content. Within the inner circle, colored sections represent synteny blocks between two version genomes. All distributions are generated using a window size of 100 Kb

In this study, we first selected a white cultivated *L. maritima* individual and performed PacBio HiFi and Hi-C sequencing to assemble a high-quality genome that would serve as a solid reference for subsequent analysis. We gathered wild *L. maritima* samples from documented populations across several countries in the Western Mediterranean Basin (France, Morocco, Spain, and Tunisia), while representative selection of the cultivated varieties was purchased online. We carefully selected 41 wild individuals from 13 localities across its natural distribution range and 43 cultivated samples across most varieties for genome resequencing. Our primary objective was to investigate the genetic relationships among wild populations and between them and cultivars to trace the domestication history of this species. Specifically, we aimed to determine the domestication timeline for flower color in cultivated varieties. Finally, we integrated genomics, transcriptomics, and resequencing analyses to elucidate the genetic basis of purple flower coloration. All of these results will provide important genetic insights into future breeding of *L. maritima*.

**Table 1:** A comparison of two *L. maritima* genome assemblies.

**Assembly feature**	**LmG v1.0**	**LmG v2.0 (this study)**
**Total length of assembly**	197.77 Mb	284.31 Mb
**Number of chromosomes**	12	12
**Total length of chromosomes**	174.59 Mb	234.00 Mb
**Scaffold N50**	14.94 Mb	20.16 Mb
**Scaffold L50**	7	7
***N* Length**	5 904 383 bp	6600 bp
**Number of gaps**	5076	141
**Number of contigs**	32 810	769
**Contig N50**	0.92 Mb	6.76 Mb
**Contig L50**	533	15
**BUSCO score for assembly**	99.0%	99.2%
**LAI score for assembly**	15.41	24.07
**Number of genes**	25 813	33 960
**BUSCO score for predicted gene models**	97.1%	98.8%
**Mean gene length**	2431.12 bp	1988.35 bp
**Mean CDS length**	240.63 bp	234.89 bp
**Mean exon length**	295.55 bp	254.90 bp
**GC content**	34.95%	37.03%
**Repeat content**	41.94%	51.61%

## Results

### Genome assembly, annotation, and comparison of different assembly versions

We selected an individual of the cultivar ‘Wonderland White’ of *L. maritima* (Sample ID: P004H001, Fig. 1A and B) and generated 67.5 Gb of data totally for *de novo* genome assembly (Table S1). A total of 43.8 Gb of data was acquired from PacBio HiFi sequencing. The assembled genome size was 284.31 Mb, and the contig N50 size was 6.76 Mb. After that, we utilized Hi-C reads to anchor these contigs onto 12 chromosomes using YaHS [24]. The chromosome-scale genome assembled measured 234.00 Mb in length, with a chromosome N50 of 20.16 Mb (Table 1, Fig. S1). According to previous research [25], the estimated genome size of *L. maritima* was around 225–264 Mb. The genome assembly completeness was evaluated using BUSCO, with results indicating that 99.2% of conserved BUSCO proteins were detected (Table 1). A total of 33 960 genes were predicted, with gene prediction showing 98.8% coverage of complete BUSCOs (Table 1). It is noteworthy that the LTR Assembly Index (LAI) of our assembled genome achieved a score of 24.07, surpassing the threshold of 20, which qualifies it as a gold standard assembly. This indicates the high contiguity and completeness of our assembly.

**Figure 2:**
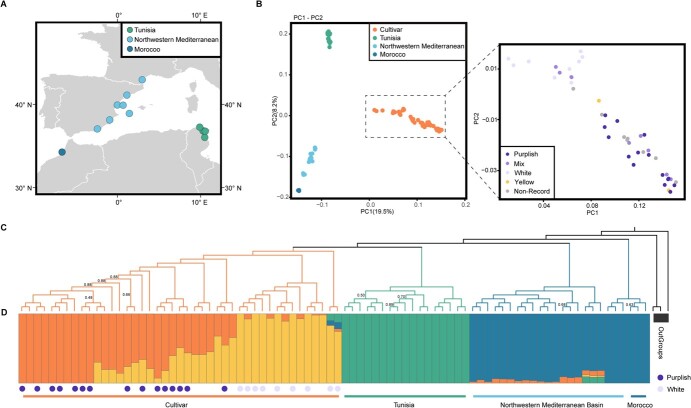
Population structure of *L. maritima*. (A) Geographic distribution of the analyzed samples, with color codes representing their various localities. (B) PCA of genome-wide SNP data, displaying the first two principal components. Colors represent phylogenetic groupings and flower colors. (C) Neighbor-joining phylogenetic tree based on SNPs, using two samples of *L. libyca* and one sample of *L. canariensis* as outgroups. Branch supports below 95% are indicated in the fig. (D) ADMIXTURE analysis with the optimal *K* value (*K* = 4), which minimizes cross-validation error. Each vertical bar represents an individual sample, with the *x*-axis denoting sample localities. Colors within the bars indicate putative ancestral backgrounds, while the *y*-axis quantifies the proportion of ancestry. The dots color indicates flower color of cultivar samples

Furthermore, we compared this new assembly (LmG v2.0) with previous *L. maritima* genome assembly (LmG v1.0) [25]. In comparison with earlier assembly based on Illumina and HiC (LmG v1.0), our innovative LmG v2.0 captured a greater number of sequences and featured an enlarged chromosome assembly size. To examine the expansion of the genome assembly in LmG v2.0, we conducted a direct nucleotide alignment using Minimap2 [26], identifying sequences unique to LmG v2.0 that were absent in LmG v1.0. This analysis revealed 84.79 Mb of additional unaligned sequence, largely accounting for the size difference. Of these sequences, 62.82 Mb (74.08%) consist of repetitive elements, while 4.71 Mb (5.56%) represent gene regions, including 3190 genes (Table S2). Notably, Chr4 and Chr8 exhibit the highest proportions of repetitive elements, at 89.35% and 82.90%, respectively, which significantly contribute to the expansion of the LmG v2.0 genome assembly. Both assemblies exhibited good collinearity, with consistent gene distribution and GC content (Fig. 1C). LmG v2.0 demonstrated a reduction in fragmentation, as substantiated by the quantity and N50 length of contigs, and notably enhanced sequence contiguity by 42.67- and 7.35-fold. These results suggested that our newly assembled LmG v2.0 possesses a longer chromosomal length, improved continuity, a higher LAI, more complete BUSCOs, and overall superior genome quality (Table 1). Therefore, LmG v2.0 provides us with a good reference for population genetics studies.

### Population genetic analyses and identification of groups and subgroups

Our dataset includes 41 wild *L. maritima* individuals across its natural distribution range and 43 cultivars (Figs 1A and 2A, Tables S3–S4). The total genome re-sequencing data were 661.9 Gb for all samples, achieving an average sequencing depth of approximately 34.6× per accession (Table S5). Reads were mapped to the LmG v2.0 high-quality reference genome, each sample exhibited an average mapping rate of around 95.8% (Table S5). Variant detection and filtering revealed 1 883 074 putative single nucleotide polymorphisms (SNPs) across all samples, which were revealed for further analyses.

Genetic relationships among wild and cultivated individuals were initially reconstructed using neighbor-joining method, principal component analysis (PCA), and ADMIXTURE (Figs 2B-D and S2-S3). The PCA separated wild and cultivar samples along PC1 (explaining 19.5% of variance). Further clustering divided the samples into three groups: the wild TS group from Tunisia, the wild NM group from Morocco and the Northwestern Mediterranean Basin (i.e. French and Spanish populations), and the CU group for all cultivars (explaining 8.2% of variance) (Fig. 2B). As for the CU group, upon closer examination, this subset of individuals was further divided into two distinct groups according to flower color: white and purplish. Other individuals with different flower colors were mixed within this subset. Admixture analysis of the genetic structure of all *L. maritima* yielded results consistent with those from PCA. Model complexity that minimizes cross-validation (CV) error is 4 (CV error = 0.3773; Figs 2D and S3). Wild and cultivar samples were clearly separated with *K* = 2. Wild *L. maritima* from different geographical locations were further clustered into two genetic groups when *K* = 3. Notably, at *K* = 4, which showed the lowest CV error, two distinguishable components emerged within the cultivated populations, predominantly correlating with variations in flower color. Specifically, all individuals with white flowers showed a high proportion of a single component, with other components comprising less than 10% (Fig. 2D). The phylogenetic analysis based on SNPs also grouped all samples into distinct genetic clusters (Fig. 2C). Group NM was first diverged while Group TS and CU followed. Our analyses identified four distinct genetic clusters within *L. maritima*, comprising two wild groups and two cultivar groups. On the one hand, Group TS and Group NM predominantly mirrored their respective geographic distributions. On the other hand, the two components within Group CU were correlated with distinct flower colors.

**Figure 3:**
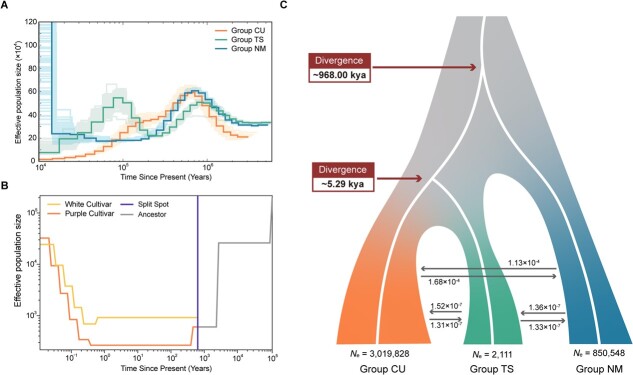
Demographic history of different genetic groups of *L. maritima*. (A) Demographic history inferred using the PSMC method. (B) Demographic history inferred using the SMC++ method. (C) Graphical summary of the optimal demographic model determined by fastsimcoal2

### Genetic diversity and demographic history of each genetic group

The cultivated CU group exhibited the lowest genetic diversity (3.13 × 10^−3^), while the two wild groups, TS and NM, showed a higher diversity (6.63 × 10^−3^ and 1.07 × 10^−2^, respectively). Additionally, genetic differentiation (*F*_ST_) value showed the highest differentiation between the TS and CU groups, followed by NM and CU, with the lowest differentiation observed between the two wild *L. maritima* groups, TS and NM groups (Fig. S4).

We then applied pairwise sequentially Markovian coalescent (PSMC) [27] modelling to reconstruct the population-size changes history of *L. maritima*. All three groups showed comparable declines in effective population sizes since 800 000 years ago, likely reflecting their common ancestry prior to divergence. Subsequently, these groups experienced population increases, albeit at different times (Fig. 3A). Group TS had the lowest effective population size during the decreasing phase, which then the population size began to increase around 200 000 years ago and continued until about 90 000 years ago. Meanwhile, Group CU showed a modest increase from approximately 200 000 years ago up to 100 000 years ago. In contrast, the effective population size of Group NM continued to decrease throughout the period since 800 000 years ago, with only recent signs of stabilization or potential expansion (Fig. 3A).

To further investigate the demographic changes between samples with different flower colors in Group CU, we first selected two cultivar samples (one white and one purple) to analyze changes in their effective population sizes using PSMC modelling. The results showed that cultivars with different flower colors exhibited similar trends in effective population sizes (Fig. S5), suggesting that two types of individuals with purple and white flower color share the common ancestor and their divergence occurred relatively recently. However, since PSMC provides poor estimates of recent population sizes due to limited recombination and coalescence information from a single genome [28, 29], we used SMC++ [30] to also explore the demographic history changes of purple and white *L. maritima* populations. According to the results of SMC++, these two groups likely split approximately 700 years ago. After divergence, the population with purple flowers initially had a very low effective population size but began to expand in recent times (Fig. 3B).

**Figure 4:**
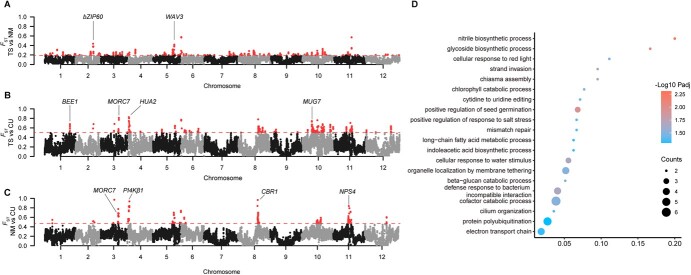
Genetic diversity of each genetic group of *L. maritima*. (A) Population divergence (*F*_ST_) between TS and NM. (B) Population divergence (*F*_ST_) between TS and CU. (C) Population divergence (*F*_ST_) between NM and CU. The dotted line represents the threshold for the top 1% of *F*_ST_. (D) GO enrichment of genes under selection between TS and CU

### Phylogenetic relationships and domestication locality

We first used SNPs from biparentally inherited nuclear genomes and assemblies of maternally inherited plastomes to examine phylogenetic relationships of all sampled individuals. We found that nuclear phylogeny was basically congruent with population genetic analyses except for that two subgroups of the Group NM did not comprise one monophyletic clade (Figs 2C and S6). However, the plastome phylogeny of all subgroups was incongruent with the nuclear tree in the phylogenetic relationships of subgroups that may result from incomplete lineage sorting and/or hybridization-triggered plastome introgression (Fig. S6). However, all analyses suggested that all cultivars comprised a well-supported lineage, nested deeply within the Group TS. This indicates that this plant might be domesticated from the wild populations near Tunisia.

We further used fastsimcoal2 to examine divergence times and potential gene flow between the cultivated lineage and the two wild lineages. Among the 11 migration models evaluated (Fig. S7), the model with the highest Akaike’s weight value was determined to be the best fit (Table S6). This best-fit model (model 5) indicated that gene flow occurred between all three groups after their initial divergence, although it was relatively low (highest at 1.68 × 10^−4^, Fig. 3C). Our model also suggested that Group NM first separated from the other two groups around 968 thousand years ago (kya), while Group TS and Group CU diverged from each other approximately 5.29 kya. This result is consistent with the phylogenetic inference. Additionally, Group CU currently has the largest effective population size (*N_e_*) among the three groups (Fig. 3C).

### Genetic divergence between wild and cultivated groups

To further explore the differentiation between the three groups, we calculated the population *F*_ST_ index to identify genes with high divergence between Group TS and Group NM. We designated candidate selective regions as those within the top 1% of *F*_ST_ windows, which identified 233 windows fulfilling this criterion (Fig. 4A). Within these regions, we identified a total of 336 genes, with those showing greater differentiation potentially linked to the adaptation of these groups to distinct environments. Notably, *bZIP60*, which was identified under selection, has been confirmed to be associated with the ER stress response [31, 32]. Under ER stress conditions, *bZIP60* functions in sensing ER stress, undergoing cleavage, and translocating to the nucleus, thereby initiating downstream gene expression [33]. Meanwhile, *WAV3*, who regulates auxin transport from shoots to roots to facilitate environmental adaptation responses to obtain the greatest possible growth advantage [34–36], was identified as a candidate selection gene. Moreover, the gene ontology (GO) enrichment shows that these candidate genes found in the top 1% of *F*_ST_ windows are associated with functions such as defense response to bacteria, regulation of response to oxidative stress, ER transport, and organization (Table S7).

We also detected 233 candidate selection windows between Group TS and Group CU, identifying a total of 313 genes showing greater differentiation that may have been selected during the domestication process (Fig. 4B). The GO functional enrichment shows that most genes are involved in glycoside biosynthetic process, nitrile biosynthetic process, and positive regulation of seed germination (Fig. 4D, Table S8). Additionally, we discovered a group of flowering-related genes that exhibited significant differentiation between the two populations (Table S9). Their functions have been reported in many studies [37–43]. The differentiation of these flowering-related genes, along with their involvement in various biosynthetic processes, likely played a crucial role in the domestication of *L. maritima*, enhancing its flowering traits and adaptability.

Population divergence between Group NM and CU revealed 233 windows within the top 1% of divergence values, encompassing 87 genes (Fig. 4C). These genes exhibit substantial differentiation, suggesting selection pressures that may drive unique environmental adaptations in each group. For instance, *CBR1* is associated with pollen functionality and seed maturation, exhibiting significant divergence. Additionally, *NPS4* and *PI4Kβ1*, both of which play crucial roles in responses to biotic and abiotic stresses, were identified as divergent. The functions of these genes in stress response pathways further underscore their potential contributions to the adaptive differentiation observed between the two populations. GO enrichment analysis further suggests that these genes are related in DNA integration, pathogenesis, and regulation of histone H3-K9 methylation (Table S10).

### Genetic changes contributing to purple flower color in cultivars

Wild *L. maritima* exclusively bears white flowers in its natural state. In contrast, cultivated *L. maritima* displays a variety of flower colors, especially purple. To explore whether anthocyanins contribute to the purple color in cultivated *L. maritima*, we first quantified the anthocyanin content in white and purple flowers. We observed notable differences in anthocyanin levels between white and purple flowers (Fig. S8). These findings highlight a clear variation in pigment accumulation between the two flower color types.

Given the extensive research on the anthocyanin pathway in plants, we selected anthocyanin pathway genes previously reported within the Brassicaceae family as references [44, 45]. Our analysis confirmed the presence of anthocyanin pathway-related genes in both versions of the LmGs. The results showed that both genomes contain a similar number of genes associated with the anthocyanin pathway. For highly conserved core genes of this pathway (such as *CHS* and *CHI*), the analysis of both genomes produced consistent findings (Table S11). These findings imply that the color variation between white and purplish flowers in *L. maritima* may be attributed to regulatory mechanisms, such as transcription factors, genetic variation, or other factors, rather than gene duplication or loss.

Additionally, transcriptome sequencing was performed on five individuals each of white and purple *L. maritima*. Subsequently, we normalized the obtained sequencing data and identified differentially expressed genes (DEGs) across various tissues. Combining these results with the previously identified anthocyanin pathway-related genes, we observed differential expression in numerous genes within the anthocyanin synthesis pathway, including several key genes (such as *CHS*, *DFR,* and *UFGTs*; Fig. S9). This complexity makes it challenging to directly attribute the cause of purple flower color solely to transcriptome analysis.

**Figure 5:**
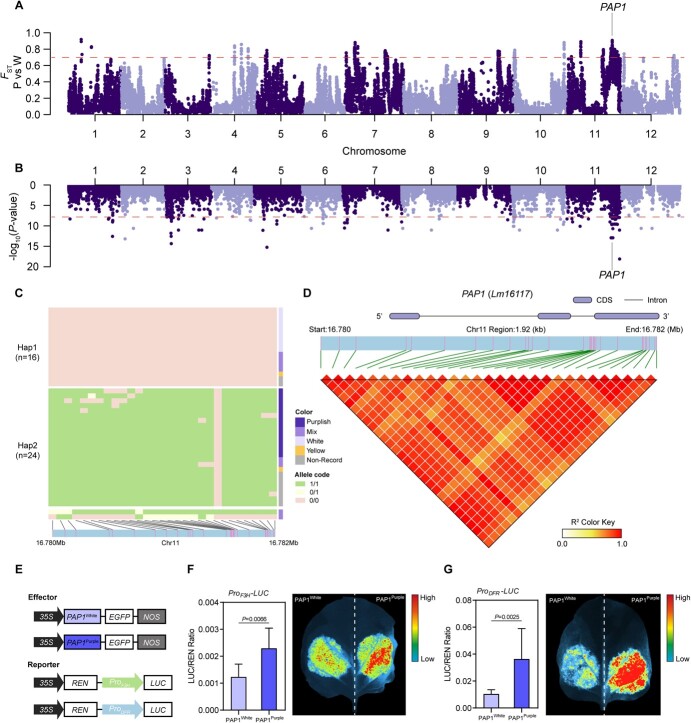
Multiple analyses identified the gene responsible for purple flower color in *L. maritima*. (A) *F*_ST_ analysis between white and purple cultivar groups. The dotted line represents the threshold for the top 1% of *F*_ST_. (B) GWAS analysis of flower color traits across the genome. The dotted line represents the threshold for genome-wide significance in GWAS. (C) Haplotype analysis of the candidate gene *PAP1*(*Lm16117*) in different color cultivar samples. The two major haplotypes, Hap1 and Hap2, are shown along with their distribution among the samples. (D) Heat map of SNP markers in LD with the most strongly associated SNPs within the PAP1 region on chromosome 11 (from 16.780 to 16.782 Mb). The color key represents the R^2^ value, indicating the strength of LD. (E) Constructs of effector and reporter genes used to validate the function of PAP1 haplotypes. The effector constructs include *PAP1* with either the Hap1 (White) or Hap2 (Purple) variant, both driven by the 35S promoter. The reporter constructs include a LUC gene driven by a promoter responsive to PAP1. (F, G) LUC/REN ratio indicating the activity of the PAP1^White^ effector in comparison with the PAP1^Purple^ effector in the LUC assay for the F3H and DFR, respectively. Significant differences are noted in the figure.

We further conducted both *F*_ST_ analysis and genome-wide association study (GWAS) to investigate the potential causes of purple flower color in population data. Initially, we identified 769 genes within 233 candidate selection windows (the top 1% of *F*_ST_ windows, Fig. 5A). Functional enrichment analysis using GO revealed that the candidate genes are associated with various functions, including adaptation to biotic and abiotic stress, involvement in biosynthetic processes, and regulation of the anthocyanin metabolic pathway (Table S12).

Furthermore, we identified 885 candidate genes that showed association to flower color by GWAS analysis. We further selected the most significant associations with the target trait and multiple SNPs corresponding to the same gene as the target SNPs. These SNPs corresponded to genes located on chromosome 11 (Fig. 5B). Among these genes, nine were also detected by the selective scan, and three of them located on chromosome 11 were further investigated in detail as potential contributors to the formation of purple flower (Table S13). Finally, we identified *Lm16117* as an orthologue of the *PAP1* in *A. thaliana*, which is a crucial transcription factor involved in regulating the anthocyanin pathway [13, 46, 47]. The *PAP1* gene haplotype distribution was consistent with flower color phenotype. Specifically, all *L. maritima* individuals with white flowers carried the *PAP1*-Hap1 haplotype as a pure genotype whereas all individuals with purple flowers carried the *PAP1*-Hap2 haplotype as the other pure genotype, and those with mixed-color flowers exhibited a distribution of both haplotypes as heterozygous genotypes (Fig. 5C). We found that all wild samples of this species had the *PAP1*-Hap1 haplotype, which was consistent with their recorded colors (white in all instances). The variation between two haplotypes mainly resides in exon 3 of *PAP1*, leading to amino acid changes in the PAP1 protein that may influence its structure or function (Fig. S10). Additionally, we detected strong linkage disequilibrium (LD) on chromosome 11 from 16.780 to 16.782 Mb (Fig. 5D). In the population with purple flowers, this region also exhibited lower genetic diversity (π) values and lower Tajima’s *D* values (Fig. S11), all indicating that *PAP1* is under selection in the population with purple flowers.

To further explore the regulatory role of *PAP1* in the formation of purple flower from the wild ancestor, we employed the luciferase (LUC) reporter assay system to investigate the functional differences of PAP1 haplotypes between different flower colors (Fig. 5E). We selected *F3H* (flavanone 3-hydroxylase, *Lm01779*), *DFR* (dihydroflavonol 4-reductase, *Lm23634*), and *UFGT* (UDP-glucose3-O-glucosyltransferase, *Lm03960*) to assess the transcriptional regulation of PAP1 in different flower colors (Table S11). Through dual-LUC reporter assays system, we observed PAP1 from purple flower samples had significantly higher activation abilities on both *F3H* and *DFR* promoters than those from white, indicating that PAP1 from purple flowers had a more pronounced regulatory effect on these genes (Figs 5F-G and S12). Regarding *UFGT*, no significant difference in LUC activity was observed between the two different haplotypes of PAP1 (Fig. S12).

## Discussion

In this study, we created a high-quality reference genome for *L. maritima* with a total length of 284.31 Mb, using a combination of PacBio HiFi and Hi-C sequencing data. Our population genetic analyses included 84 *L. maritima* samples, covering both wild and cultivated populations. We identified three genetic groups: two wild and one domesticated. We found that the wild ancestor of all cultivated *L. maritima* varieties may be near Tunisia (northern Africa). One wild group did not contribute genetically to the current cultivars. Furthermore, we conducted extensive population genomic analyses on cultivated *L. maritima* with different flower colors using multiple methods, ultimately determining the genetic changes responsible for the purple flower coloration from the ancestral pure white flowers. These findings enhance our understanding of the genetic mechanisms underlying flower color in the ornamental *L. maritima* and establish a foundation for future breeding efforts, including the introduction of genetic resources from another wild group.

### Local adaptation of wild groups and domestication location of the cultivars

The wild *L. maritima* populations were recorded to occur around the Mediterranean coast [17, 18]. Population genetic analyses indicate that all sampled natural populations were classified into two distinct lineages: the NM group occurring in Morocco, Spain, and Mediterranean France, and the TS group in Tunisia (Fig. 2). Population demographic history revealed that the NM and TS groups experienced similar population contractions. Notably, the TS group showed a significant population expansion around 200 000–90 000 years ago (Fig. 3), likely due to climatic factors such as changes in precipitation and temperature patterns linked to the transition from the Penultimate Glacial Period (PGP, ca. 135 000–195 000 years ago) to the Last Interglacial (LI, ca. 115 000–130 000 years ago). The warmer and, particularly, more humid conditions at the LI compared to the PGP could explain such population expansion (it was a peak of increased humidity in the Sahara between 92 000 and 129 000 years ago) [48]. Fossil records and paleoclimatic studies indicate that North Africa, including Tunisia, underwent significant climatic fluctuations during this period, which is known to have influenced the distribution and evolution of many plant species [48–50]. These Pleistocene climatic changes likely resulted in fluctuations of the population sizes of the wild *L. maritima* populations. In particular, we found that the two major wild groups experienced local adaptation with high differentiation in genes related to habitat responses, including defense response to bacteria, regulation of response to oxidative stress, and endoplasmic reticulum transport and organization (Table S7).

All cultivars were found to comprise a single monophyletic lineage (Fig. 2C). This cultivar lineage is nested within the TS group based on maternally inherited plastomes (Figs 2C and S6), suggesting that all cultivars of *L. maritima* might have been domesticated from one wild population near Tunisia. The admixture analyses also suggested that the wild TS group was mixed by the cultivar CU group and the wild NM group (Fig. S3) when *K* = 2. This structure would have been formed by the strong founder effects experienced by the cultivated CU group when arose from its ancestor (the TS group), from which only a few alleles were selected. During domestication, the selected optimal alleles were mainly related to those genes involving in glycoside biosynthetic process, nitrile biosynthetic process, and seed germination (Fig. 4C and Table S8), all of which are essential for plant cultivation practices by humans [51, 52].

Throughout domestication, numerous genes have come under selection. Using the population *F*_ST_ analysis, we identified a total of 400 genes showing signs of selection between the wild and cultivated group, most of which are implicated in environmental adaptation (Fig. 4). Notably, a group of flowering-related genes displayed significant differentiation between Group TS and CU, with previous studies frequently highlighting their roles (Table S9). *HUA2*, a component of the floral homeotic *AGAMOUS* pathway, is essential in *Arabidopsis thaliana* for the proper expression of *flowering locus C* (*FLC*) and *agamous* [37]. These genes are crucial regulators of flowering time and reproductive development, respectively. Single *hua2* mutants exhibit early flowering and reduced levels of *FLC* mRNA [37, 38]. Additionally, *HUA2* plays a role in co-regulating flowering and anthocyanin biosynthesis under high light and low-temperature stress [39]. Similarly, *BEE1* enhances flowering through its interaction with *FT*, while acting as a negative regulator in abiotic stress responses [40–42]. *MORC7* can suppress *FWA* expression, thereby promoting early flowering [43], and *MUG7* is involved in the stress response and floral development processes [53]. Collectively, these genes likely contribute to the enhanced reproductive and stress resilience traits observed in cultivated populations, illustrating the selective pressures and adaptive evolution that have shaped *L. maritima* over time.

In addition, the divergence of the CU cultivar lineage from the closely related TS subgroups was estimated to have occurred approximately 5290 years ago (Fig. 3C), indicating domestication of sweet alyssum during the middle Holocene. This aligns with archaeological evidence that early Neolithic societies in northern Africa, capable of agriculture and animal domestication, were established around 7000 years ago [54]. Although the specific archaeological record in Tunisia is sparse for the last 6000–5000 years BP [55], rock paintings in Jebel Ousselat [56] might indicate the presence of agricultural societies during this period. Such early human activity likely influenced the selection and propagation of *L. maritima*, as these communities could have domesticated plants for various uses. It is interesting that we found the cultivars in the recent past showed weak or little gene flow with the NM wild group although genetic changes between cultivars and the ancestor TS group occurred.

Building on this context, our estimates indicate that a sub-lineage with purple flowers likely originated around 700 years ago (Fig. 3B), coinciding with the European Late Middle Ages or Proto-Renaissance period in the 14th century. Although some plants, such as *Rosa*, are reported to have undergone secondary domestication at this time [57], it is likely that the sub-lineage with purple flowers originated within the Muslim-ruled territories of Spain (Al-Andalus) or northern Africa. These regions were known for their advanced horticultural techniques. For example, the gardens and orchards of Al-Andalus were responsible for introducing, domesticating, and dispersing many Eastern crops into Europe [58, 59], including numerous ornamental plants [60]. These advancements likely promoted domestication, widespread distribution, and diversification of flower colors in sweet alyssum. Therefore, climate change and human activities appear to have jointly influenced the origin, spread, and domestication of sweet alyssum. However, the genetic resources of the wild NM group should be utilized in the future breeding of this ornamental plant.

### Genetic bases for development of purple flower color after domestication

The variation in flower colors in *L. maritima* is primarily governed by anthocyanins, which are the pigments responsible for white, purple, and the mixed hues [7]. PAP1, as the main component of the MBW complex that controls the expression of genes involved in anthocyanin synthesis, has been shown to be essential for anthocyanin regulation in various plants, including *Arabidopsis*, tobacco, and banana [13, 61]. In *A. thaliana*, overexpression of *PAP1* enhances the expression of many genes, such as *PAL*, *CHS*, *DFR*, and *GST*, and then leading to the accumulation of anthocyanins [13]. Additionally, during seed development, PAP1 enhances anthocyanin accumulation by directly upregulating the expression of key genes such as *ADT5*, *CHS*, *F3H*, *DFR*, *ANS*, *3GT*, *UGTs*, and *GST* [62]. Here, we used *F_ST_* analysis and GWAS to explore highly divergent genes between cultivars with white and purple flowers. We identified two distinct haplotypes of the *PAP1* gene in *L. maritima* that are associated with white and purple flowers (Fig. 5). We performed LUC reporter system experiments to confirm that two types of PAP1 alleles differentially regulate the targeted genes *CHS* and *DFR*, leading to the color differences observed in the white and purple individuals (Fig. 5). Furthermore, we found that the *PAP1* haplotype associated with the purple phenotype was only present in the cultivated sweet alyssum, while the other *PAP1* haplotype associated with the white flower was found in both natural and cultivated populations. This suggests that domestication and artificial selection may have promoted the origin of the purple-associated *PAP1* haplotype and the new purple phenotype.

Mutations in other transcription factors that control anthocyanin accumulation, for example, the *MYB* genes, have been widely reported to affect flower color and other phenotype traits. For instance, mutations in *AbMYB1* in *Atropa belladonna*, *MdMYB10* in apple, and *CsMYB75* in purple tea have been found to affect anthocyanin production and accumulation [63–65]. These findings, together with our detailed analysis of the *PAP1* gene, highlight (1) the importance of transcription factors in regulating anthocyanin biosynthesis across various plant species, and that (2) their mutations may produce diverse phenotypes related to color changes.

While our LUC assay provides substantial evidence for the regulatory role of PAP1 in the anthocyanin biosynthesis pathway in *L. maritima*, further functional validation, such as transgenic studies, would yield additional insights into its precise role in color differentiation. However, due to the lack of an established transgenic platform in *L. maritima*, implementing these approaches remains challenging. This limitation underscores an important direction for future research, as developing such a platform would enable a more comprehensive exploration of gene functions in this species. Overall, our study provides a comprehensive genomic framework for *L. maritima*, elucidating the genetic basis of flower color variation and offering valuable resources for future genetic and breeding research. The identification of key genes and haplotypes involved in flower color regulation lays the groundwork for advanced breeding programs and enhances our understanding of the domestication and evolution of horticultural plants.

## Materials and methods

### Sampling and sequencing

The individual of *L. maritima* with white petals for genome sequencing (Sample ID: P004H001; Cultivar name: ‘Wonderland White’) was cultivated in Sichuan University, Chengdu, Sichuan Province, China (30.629°N, 104.089°E). We used the same strategy to harvest plant materials, DNA, and RNA as descripted in our previous study [66]. For PacBio HiFi sequencing, we utilized a single-molecule real-time cell on a PacBio Sequel II platform, generating HiFi reads using CCS with default settings for the sequenced samples. For RNA-seq, the RNA was sequenced on the Illumina HiSeq X Ten system using paired-end mode (2 × 150 bp). Raw RNA reads were processed to obtain clean reads by filtering out low-quality reads and trimming adapter sequences using Trimmomatic v0.39 [67] and assessed for quality using FastQC v0.11.9. Finally, the Hi-C library preparation and sequencing were performed as previously described [68].

We collected 41 wild *L. maritima* samples from 13 localities in the southwestern Europe and northern Africa for resequencing (Table S3). Wild *L. maritima* samples were collected from multiple locations: TS group from Tunisia, NM group from Morocco, and the northwestern Mediterranean Basin (including populations from France and Spain). We also collected two *L. libyca* samples (Sample ID: P059H001, P060H001, Table S3) and one *L. canariensis* sample (Sample ID: P061H001, Table S3) as outgroups. All cultivar *Lobularia* materials were grown in Sichuan University, Chengdu, Sichuan Province, China (30.629°N, 104.089°E; Table S4). Fresh leaves were preserved in silica gel for subsequent DNA extraction. Sequencing was then performed on the BGISEQ-500 platform using a 500-bp paired-end library, with a target coverage of 30×. Meanwhile, we selected one individual from each locality and sequenced them with a target coverage of 50× to ensure adequate sequencing depth and quality.

### Genome assembly, gene annotation, and synteny analysis

The first assembled contig of the *L. maritima* genome was accomplished by using hifiasm v0.19.5-r587 [69] with default settings. After that, the Hi-C reads were cleaned with Fastp v0.23.1 [70] and aligned to contigs using Burrows-Wheeler Aligner (BWA) v0.7.17 [71]. Then, YaHS v1.2a.1 [24] and Juicertools v1.19.02 [72] were used for anchoring the contigs into chromosomes and manual correction. We used BUSCO v5.4.7 [73] to assess the assembled quality with the databases ‘embryophyta_odb10’. Also, the LAI value [74] was used to evaluate the quality of the genome.

Repetitive elements in the *L. maritima* genome were identified based on methods described in previous studies [66, 75], using RepeatMasker v4.1.2-p1 [76], RepeatModeler v2.0.2a [77], LTRharvest v1.5.10 [78], LTR_Finder v1.06 [79], and LTR_retriever v1.9 [80] for annotation. The repeat library for *L. maritima* genome was constructed using EDTA [81], which combines both structure- and homology-based methods for *de novo* TE annotation.

Protein-coding genes were predicted and annotated using the GETA tool (https://github.com/chenlianfu/geta). Augustus v3.2.3 [82] was used for *de novo* gene prediction. We selected the related species *Arabis alpina*, *A. thaliana*, *Capsella rubella*, *Eutrema salsugineum*, *L. maritima* (LmG v1.0), *Megadenia pygmaea,* and *Pugionium cornutum* for a homology-based approach using GeneWise v2.4.1 [83] (Table S14). All the evidence was integrated through the GETA pipeline to produce the final gene set for the *L. maritima* genome.

The functional annotation was generated using Swiss-Prot, TrEMBL [84], and InterPro database [85], with GO terms and metabolic pathways annotated through Blast2GO v2.5 [86], eggNOG [87], and KEGG databases [88]. By integrating the annotation results identified through the methods, the final functional annotation results of the genes were obtained. MCScanX [89] and Minimap2 v2.26-r1175 [26] were used to detect collinearity between two versions of *L. maritima* genomes. With a window length of 100 000 bp, the GC content and gene density of each window were also counted and visualized using Circos software v0.69–6 [90].

### SNP calling

We used the newly assembled *L. maritima* genome as reference, mapping all samples with BWA v0.7.17 [71]. Subsequently, SAMtools v1.6 [91] was employed to calculate the mapping rate, discarding those with rates below 70%. Following this, we used the Genome Analysis Toolkit v4.4.0.0 [92] for multisample SNP and genotype calling. After obtaining the original VCF file, we used previous filtering steps to reduce false positives [93].

### Population structure analyses

We initially applied LD filtering using plink v1.90 [94] with the parameter: —indep-pairwise 100 10 0.2. We conducted a PCA using the R package ‘SNPRelate’ [95] to visualize genetic relationships among samples. Ancestry inference was performed using ADMIXTURE v1.3.0 [96] with *K* values ranging from 2 to 10. The optimal *K* was determined by cross-validation. Then, we used VCF2Dis v1.50 (https://github.com/BGI-shenzhen/VCF2Dis) to construct a neighbor-joining phylogeny with bootstrap support with its default parameters. Using GetOrganelle v1.7.7.0 [97], we assembled the chloroplast of *L. maritima* and used RAxML-NG v1.2.1 [98] to construct the plastomes phylogenetic tree. The data of *L. libyca* and *L. canariensis* were used as outgroups for both phylogenetic trees.

### Population demographic history

We selected four samples from cultivated *L. maritima* and two wild groups with higher mean coverage (P010H001, P021H001, P049H001, and P051H003) and applied the PSMC model [27] to analyze the changes in effective population size following default parameters. To convert the scaled times and population sizes into real values, we applied a mutation rate of 7.0 × 10^−9^ per nucleotide per generation [99] and set a generation time of 1 year. SMC++ [30] was also employed to estimate recent divergence times and effective population size changes, using the same mutation rates and generation times as above. Simulations were performed with SMC++ using its default parameters.

Fastsimcoal2 v2.7 (fsc27) [100] was also used to explore the demographic history of cultivated *L. maritima* and two wild *L. maritima* groups. We set a total of 11 different models to explore the best-fitting divergence model. We focused on SNPs at fourfold degenerate sites (with no missing data across all individuals) to mitigate the effects induced by selection. The other simulation parameters and methods were consistent with those used previously [93, 101, 102].

### Analysis of population genetic divergence

We utilized genomics_general (https://github.com/simonhmartin/genomics_general, accessed on 07 July 2020) to compute *F*_ST_, π, and Tajima’s *D* across various genetic groups, employing a 50-kb window and a 10-kb step size. We defined candidate selective regions as those within the top 1% of *F*_ST_ windows. And we further defined candidate genes through the following two patterns: (i) the genes should be located in candidate selective regions, and (ii) SNPs could be found in gene regions [103]. We used the *R* package topGO [104] to perform gene ontology (GO) enrichment analysis on previous defined candidate genes, with *P* values adjusted for false discovery rate (FDR). Then, we employed blastp v2.10.0+ [105] against the protein libraries of *A. thaliana*, retaining the top alignment for each gene as homologous. The annotation of each candidate gene was retrieved from The Arabidopsis Information Resource database.

### GWAS analysis

We conducted a genome-wide association study (GWAS) using GEMMA (the Genome-wide Efficient Mixed Model Association) program [106] under a mixed-linear model, incorporating the kinship (*K*) matrix to account for genetic relationships. The genome-wide significance threshold (1.47 × 10^−8^) was determined by a uniform threshold of 1/*n*, where *n* was the effective number of independent SNPs calculated using Genetic type 1 Error Calculator (v0.2) [107].

### Identification of anthocyanin pathway-related genes

All genes used in this study that are related to anthocyanin pathway have been reported in previous studies [44, 45], which included genes from both *A. thaliana* and *Brassica* species. For this analysis, we identified the corresponding homologous genes in *A. thaliana*, resulting in a total of 52 genes (Table S15). These genes were used to perform blastp v2.10.0+ [105] against the *L. maritima* protein libraries. The best match was identified as a homologous gene and used for subsequent analysis.

### Anthocyanin determination

We collected about 0.100 g of fresh white and purple *L. maritima* petal samples, with five biological replicates each, and placed them in 5 ml centrifuge tubes. Each tube was then filled with 3 ml of 1% hydrochloric acid methanol solution. The tubes were covered with aluminum foil to shield them from light and then kept at 4°C overnight for total anthocyanin extraction. After 12 h, the tubes containing the extract were centrifuged at 12 000 rpm at 4°C and the supernatant was subsequently collected for total anthocyanin content analysis. About, 200 μl of the supernatant was added to a 96-well microplate for absorbance measurement at 530 and 657 nm using a microplate reader. Finally, the anthocyanin content was calculated using the formula:


$$ \textrm{Relative anthocyanin content}= \frac{\left({A}_{530}-0.25\times{A}_{657}\right)}{\mathrm{fresh}\ \mathrm{weight}\ \mathrm{of}\ \mathrm{the}\ \mathrm{sample}}. $$

where A530 and A657 represent the absorbance values at 530 and 657 nm, respectively.

### Transcriptome analysis

To identify genes associated with flower color, the roots, stems, leaves, and flowers of white and purple *L. maritima*, each with five biological replicates, were sequenced and used for transcriptome analysis. Raw RNA reads were processed with Trimmomatic v0.39 [67] and FastQC v0.11.9 to generate clean reads. Transcript-level abundances were quantified using Salmon v1.1.0 [108] and DEGs between samples were analyzed using DESeq2 v1.22.2 [109]. Genes with an adjusted *P* value < 0.05 were considered as DEGs.

### Dual-LUC assay

We used the same protocol described previously [110] to perform transient expression and dual-LUC assays. The DNA sequences of *PAP1* from white and purple flowers, driven by the 35S promoter, served as effectors. The promoter regions of *CHS*, *DFR*, and *UFGT* were subcloned into the pGreen-0800-LUC vector as reporters. Renilla LUC (REN) in the vector served as an internal control, and the firefly to REN ratio was assessed with a dual-LUC reporter assay system. All primers for plasmid construction used in this study can be found in Table S16.

## Supplementary Material

Web_Material_uhae355

## Data Availability

All raw sequence data and genome assembly data have been deposited in National Genomics Data Center, Beijing Institute of Genomics, Chinese Academy of Sciences, and China National Center for Bioinformation, under accession number PRJCA027303.
