# Supplementary material for: Domestication history and genetic changes for the newly evolved flower color in the ornamental plant Lobularia maritima (Brassicaceae)
Source: Hortic Res. 2024 Dec 19;12(4):uhae355. doi: 10.1093/hr/uhae355 (PMC11879304; doi:10.1093/hr/uhae355)

Supplemantary Information of

Domestication history and genetic changes for the newly evolved flower color in the ornamental plant *Lobularia maritima* (Brassiaceae)

Wenjie Yang[1], Meng Liu[1], Landi Feng[1], Pengfei Jiao[1], Jiebei Jiang[1], Li Huang[1], Jianquan Liu*[1,2], Jordi Lopez-Pujol*[3,4], Quanjun Hu*[1]

[1]Key Laboratory of Bio-Resource and Eco-Environment of Ministry of Education, College of Life Sciences, Sichuan University, Chengdu 610065, China

[2]State Key Laboratory of Grassland AgroEcosystem, College of Ecology, Lanzhou University, Lanzhou, China

[3]Botanic Institute of Barcelona (IBB), CSIC-CMCNB, Barcelona 08038, Spain

[4]Escuela de Ciencias Ambientales, Universidad Espíritu Santo (UEES), Samborondón 091650, Ecuador

*Corresponding authors: huquanjun@scu.edu.cn; jlopez@ibb.csic.es; liujq@nwipb.ac.cn

Wenjie Yang: yangwj@stu.scu.edu.cn
Meng Liu: liumeng3@stu.scu.edu.cn
Landi Feng: landifeng@stu.scu.edu.cn
Pengfei Jiao: jiaopengfei@stu.scu.edu.cn
Jiebei Jiang: jiangjiebei@stu.scu.edu.cn
Li Huang: 1003882358@qq.com
Jianquan Liu: liujq@nwipb.ac.cn
Jordi Lopez-Pujol: jlopez@ibb.csic.es
Quanjun Hu: huquanjun@scu.edu.cn

Figure S1 The Hi-C chromatin interaction map for the 12 chromosomes of *Lobularia maritima.*

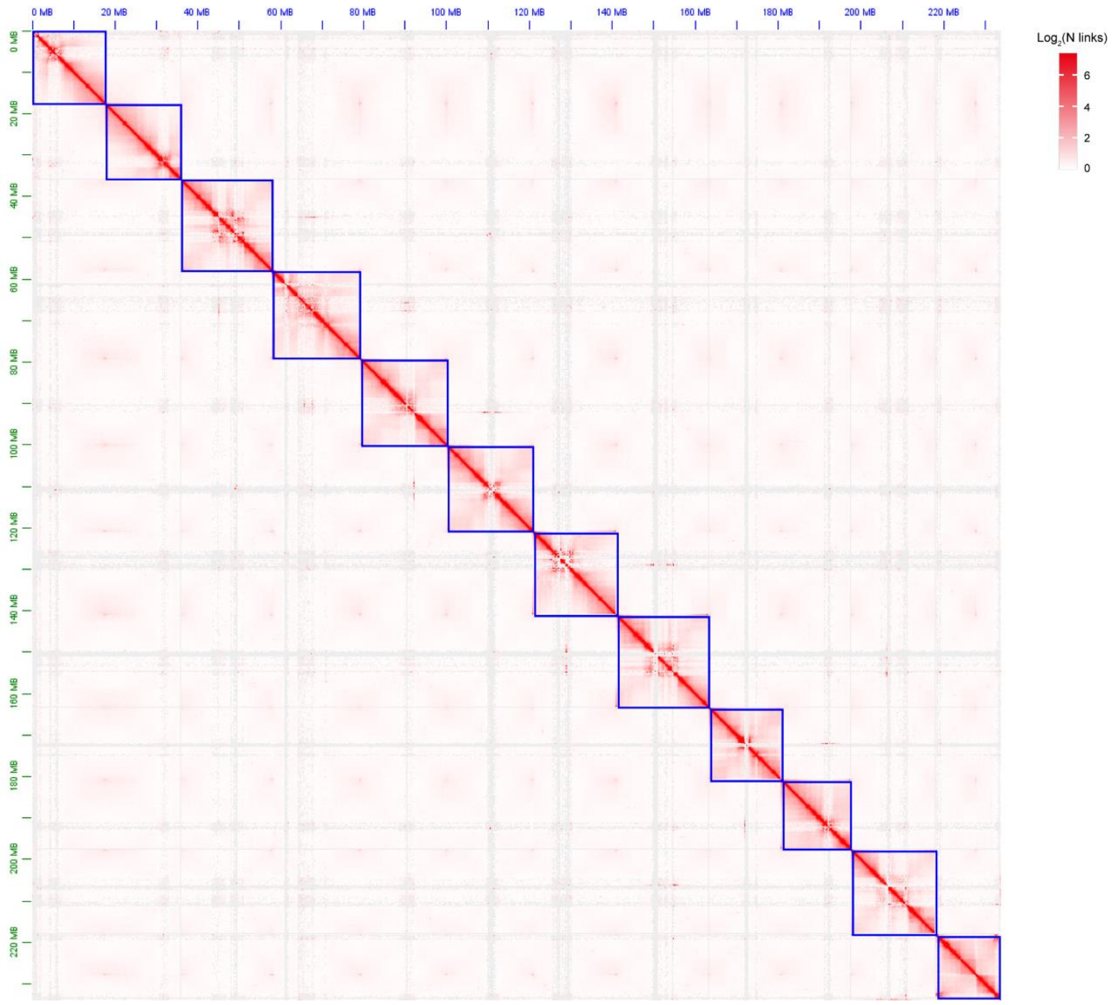

Figure S2 Principal component analysis (PCA) analysis of *Lobularia maritima* based on PC1 and PC3.

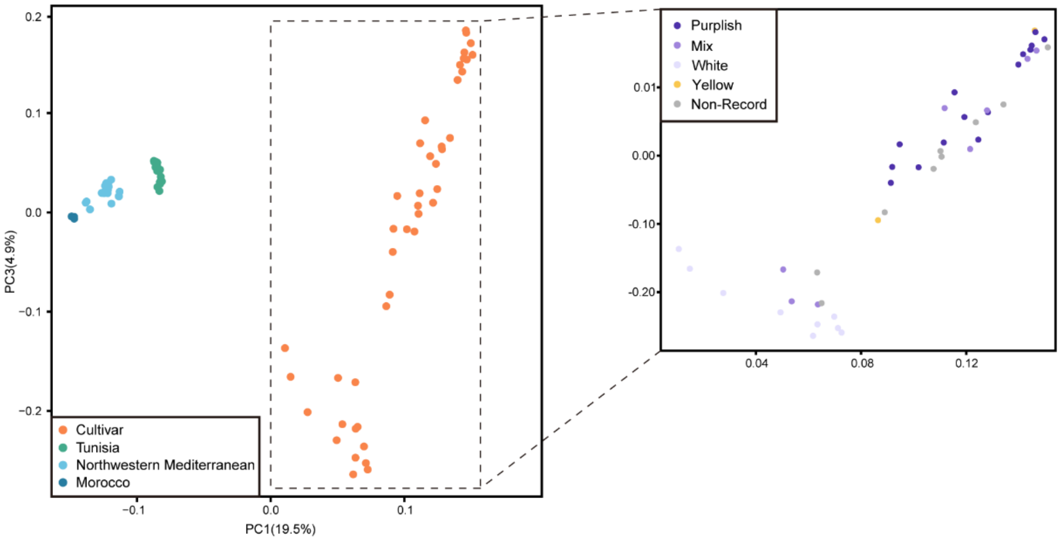

Figure S3 ADMIXTURE analysis of *Lobularia maritima*. (A) Bayesian model–based clustering analysis with different number of groups (K = 2 to 4). Each vertical bar represents one sample, and the x axis shows the four groups. Each color represents one putative ancestral background, and the y axis quantifies ancestry membership. (B) CV error value of different K.

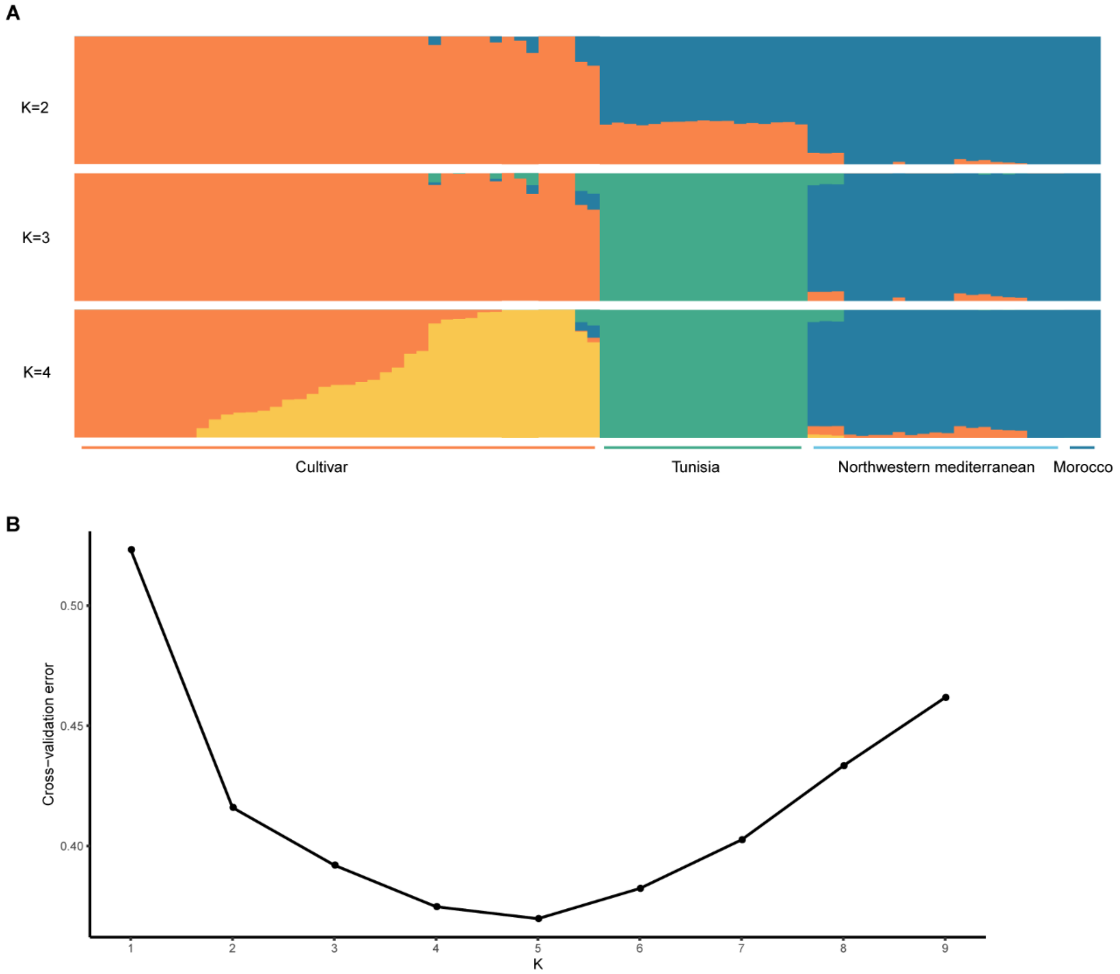

Figure S4 Nucleotide diversity and population divergence across the three groups of *Lobularia maritima*. Values in parentheses represent measures of nucleotide diversity (π) for the group, and values between pairs indicate population divergence (F_{ST}).

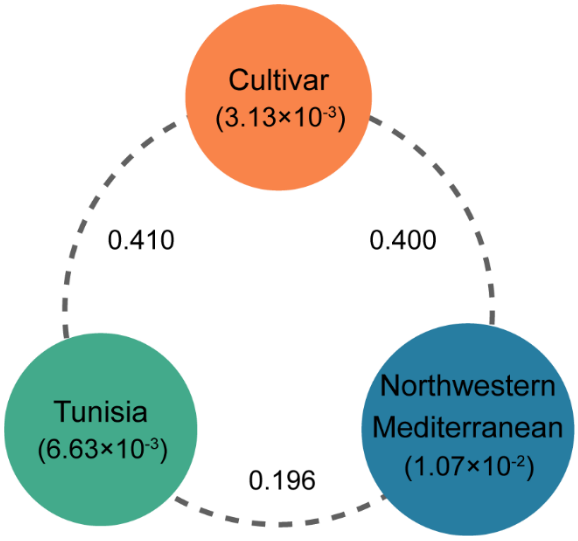

Figure S5 Demographic history inferred from the PSMC method of white and purple cultivar.

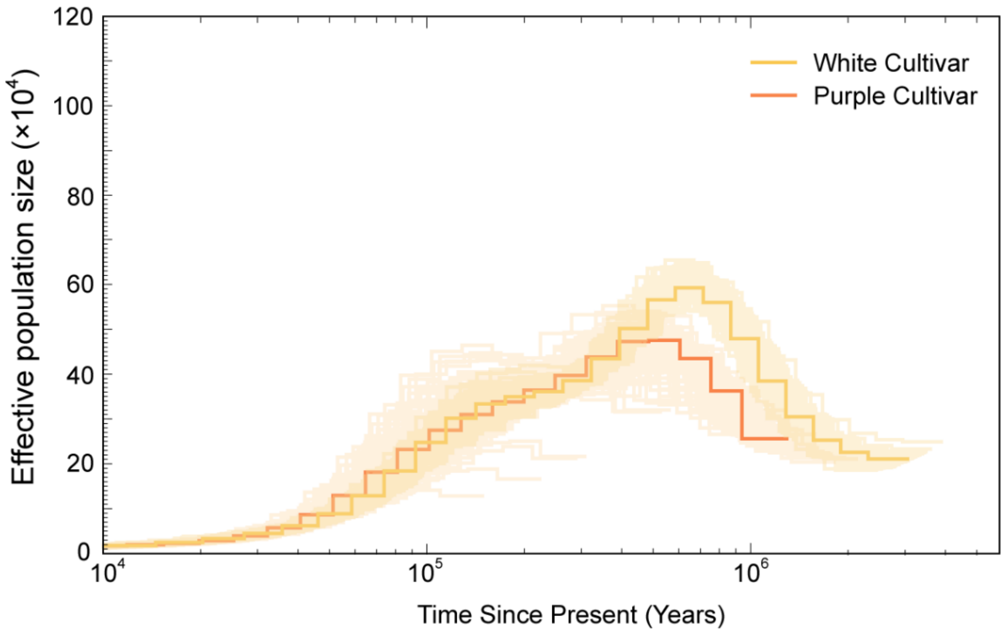

Figure S6 Plastome and nuclear phylogeny analysis of *Lobularia maritima*. Branch supports below 95% are shown in the figure.

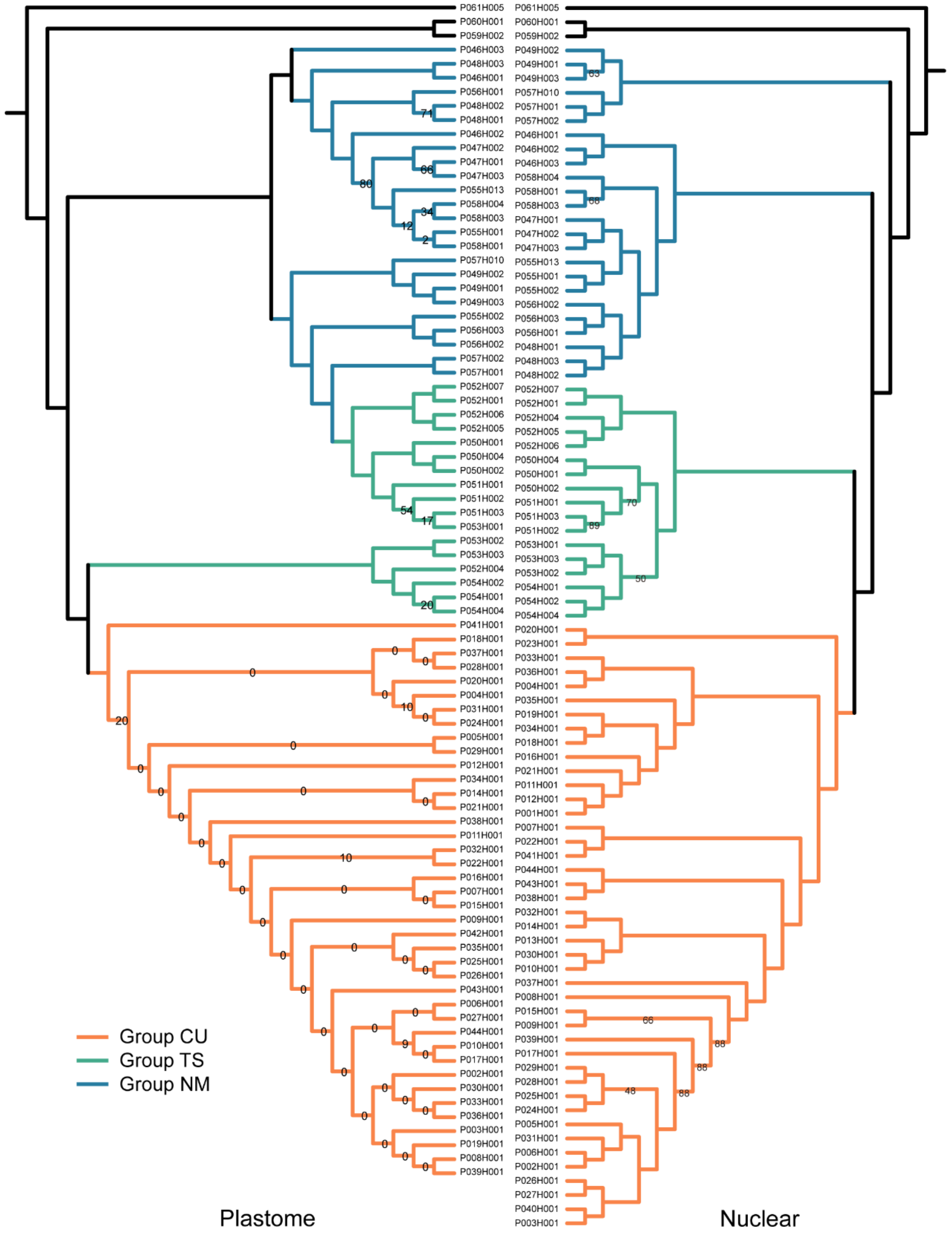

Figure S7 11 different models used in fastsimicoal analysis.

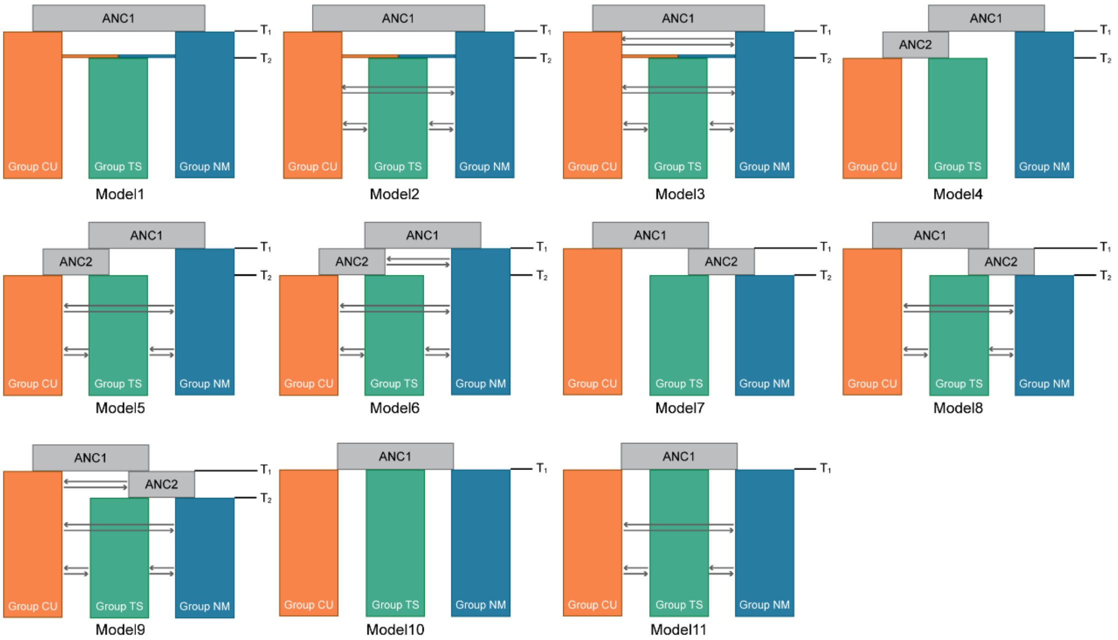

Figure S8 Anthocyanin content in white and purple flowers of *Lobularia maritima*. (A) Flowers used in this analysis. (B) Anthocyanin levels in different color flowers.

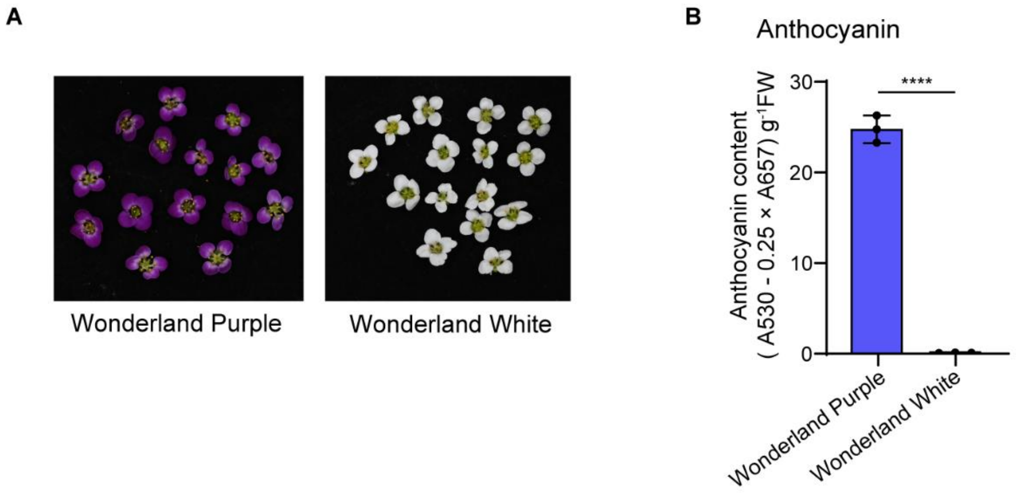

Figure S9 Differential expression of anthocyanin pathway-related genes in white and purple *Lobularia maritima*.

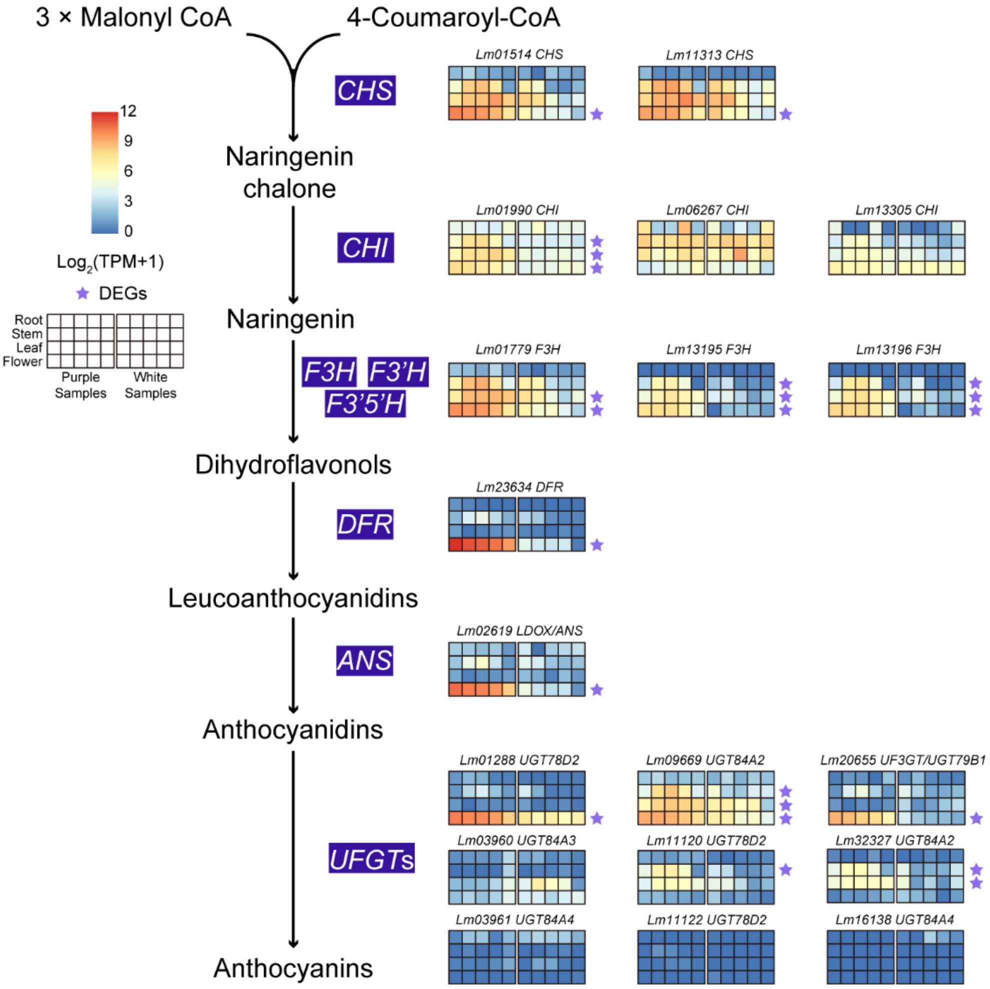

Figure S10 Variations in two haplotypes of PAP1. Lm16117 indicates the protein of *PAP1*-Hap1, Lmar022374 indicates the protein of *PAP1*-Hap2.

```
Consensus   MEGPSKGLRKGAWTAEEDSLLRQCINKYGEGKWHQVPLRAGLNRCRKSCRLRWLNYLKPC   60
Lm16117     MEGPSKGLRKGAWTAEEDSLLRQCINKYGEGKWHQVPLRAGLNRCRKSCRLRWLNYLKPC   60
Lmar022374  MEGPSKGLRKGAWTAEEDSLLRQCINKYGEGKWHQVPLRAGLNRCRKSCRLRWLNYLKPC   60

Consensus   IKRGKLSSDEVDLVLRLHKLLGNRWSLIAGRLCGRTANDVKNYWNTHLSKKXEPCCKTKM  120
Lm16117     IKRGKLSSDEVDLVLRLHKLLGNRWSLIAGRLCGRTANDVKNYWNTHLSKKQEPCCKTKM  120
Lmar022374  IKRGKLSSDEVDLVLRLHKLLGNRWSLIAGRLCGRTANDVKNYWNTHLSKKHEPCCKTKM  120

Consensus   KXXEISCTPATPVKKIDVFKPRPRSFTVXXGCXHLNGQPEDDTIXPRLGXXNXNNXXENR  180
Lm16117     KKREISCTPATPVKKIDVFKPRPRSFTVESGCSHLNGQPEDDTIPPRLGVSNNNNVYENR  180
Lmar022374  KNKEISCTPATPVKKIDVFKPRPRSFTVNNGCNHLNGQPEDDTITPRLGLNNINNFCENR  180

Consensus   ITXSKDKDVYELVDNTXDGENMWWKNLLDEXKXPDALVPZATXAEXZXTSAFDVEQLWXM  240
Lm16117     ITCSKDKDVYELVDNTLDGENMWWKNLLDECKVPDALVPEATEAEKEPTSAFDVEQLWNM  240
Lmar022374  ITSSKDKDVYELVDNTMDGENMWWKNLLDEFKDPDALVPQATAAENQATSAFDVEQLWSM  240

Consensus   LDEDXVELD                                                     249
Lm16117     LDEDDVELD                                                     249
Lmar022374  LDEDAVEL-                                                     248
```

Figure S11 π and Tajima's D around the region of *PAP1* on chromosome 11. Green box indicates the position of *PAP1* on chromosome 11.

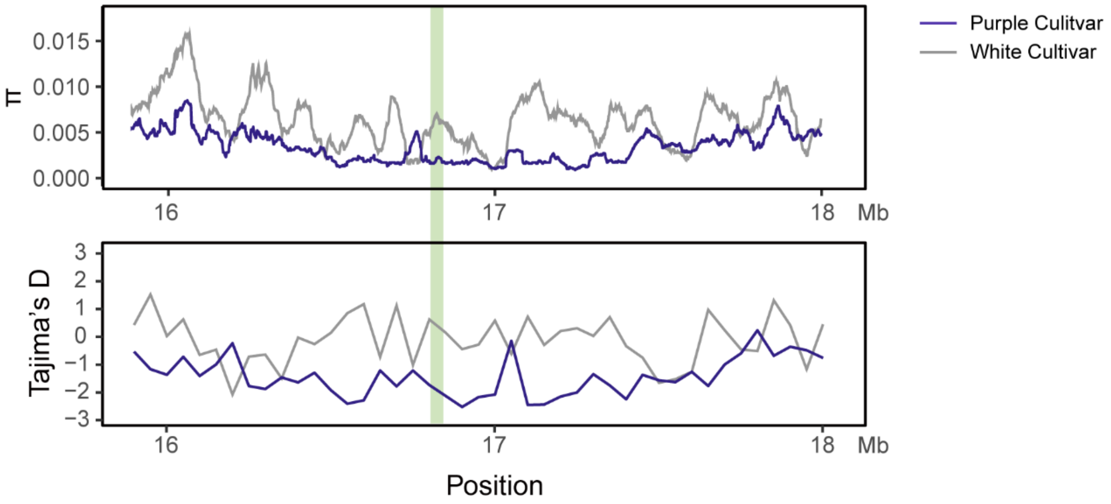

Figure S12 Luciferase (LUC) reporter gene experiment of *F3H*, *DFR*, and *UFGT*.

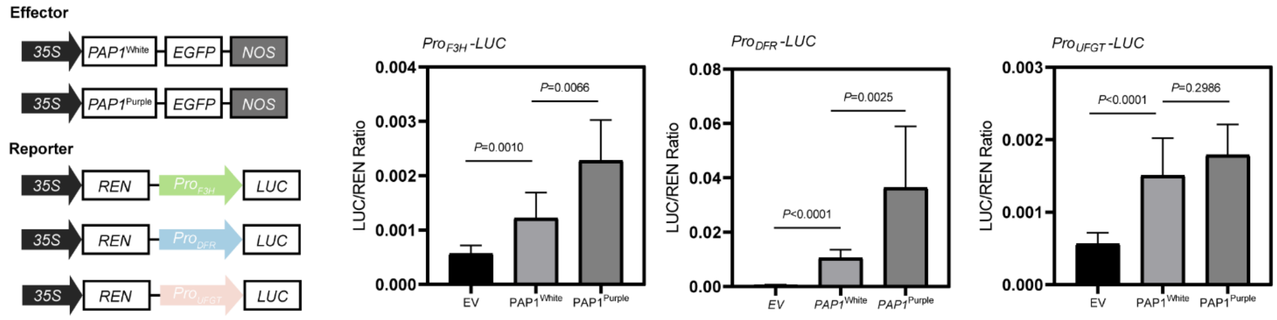

Supplement: Web_Material_uhae355 [file web_material_uhae355.zip › S_Information.pdf]
